# Stress–Strain Behaviour of Reparable Composite Panel with Step-Variable Thickness

**DOI:** 10.3390/polym13213830

**Published:** 2021-11-05

**Authors:** Andrii Kondratiev, Václav Píštěk, Lina Smovziuk, Maryna Shevtsova, Anna Fomina, Pavel Kučera

**Affiliations:** 1 Department of Building Technology and Construction Materials, O.M. Beketov National University of Urban Economy in Kharkiv, Marshal Bazhanov Str. 17, 61002 Kharkiv, Ukraine; andrii.kondratiev@kname.edu.ua; 2 Institute of Automotive Engineering, Faculty of Mechanical Engineering, Brno University of Technology, Technická 2896/2, 616 69 Brno, Czech Republic; kucera@fme.vutbr.cz; 3 Department of International Projects and Programs, National Aerospace University “Kharkiv Aviation Institute”, Chkalova Str. 17, 61070 Kharkiv, Ukraine; l.smovziuk@khai.edu; 4 Department of Composite Structures and Aviation Materials, National Aerospace University “Kharkiv Aviation Institute”, Chkalova Str. 17, 61070 Kharkiv, Ukraine; m.shevtsova@khai.edu; 5 Department of Railway, Automobile Transport and Handling Machines, Institute of Transport and Logistics, Volodymyr Dahl East Ukrainian National University, Central Avenue 59a, 93400 Sewerodonetsk, Ukraine; anyta220885@gmail.com

**Keywords:** patch, notch, transverse shear, anisotropic shell

## Abstract

There is an urgent problem of finding an economically viable method of maintenance and restoration of the bearing capacity of structures of various applications. Repair of structures with patches made of polymeric composite materials is one of the most promising repair technologies. However, an improper choice of parameters of the composite patch leads to unjustified increase in the structure mass and the cost of its further operation. These situations result from the lack of reliable methods for developing the repair process, which take into account the influence of the patch geometry and conditions for performance of repair works on the bearing capacity of the repaired structure. The mathematical model of the reparable composite shell–type panel taking into account inhomogeneity of transverse shear deformations at stepped variation of its thickness has been developed. In contrast to the classical theory of layered shells, the model allows simplifying a three-dimensional problem by setting of the displacement field on the layers’ interfaces and their linear interpolation over thickness of the panel, as well as considering the transverse shear deformations resulting from the strength, temperature, or shrinkage loading. According to results, the maximum rise in stresses in the case of a notched panel occurs in the weakened layer, and it is from this layer the failure of the structure will start. In the event of the patch, the panel surface opposite the reinforcement is the most loaded (i.e., susceptible to failure) surface. To confirm the reliability of the developed model, we compared the analytical calculations with the results of experimental and numerical studies of the deformed state of a panel of step–variable thickness by the method of holographic interferometry and modelling by the finite element method. Displacement fields available from experiments correspond to the predicted theoretical results. The resulting maximum error does not exceed 7%. The data obtained during numerical modelling allowed us to conclude that the accuracy of theoretical calculations is sufficient for engineering practice. Results of the work can be used to solve the practical problems such as determination of stress–strain behaviour of a damaged structure or structure after repair, specification of the permissible delamination dimensions, and defining of parameters of the bonded repair process.

## 1. Introduction

There is a constant need for repair technologies for constructions of various applications which do not require significant financial costs, labour and material resources [1,2,3]. From the point of view of increasing the economic efficiency of the operation of structures used in aircraft construction, rocket and space technology, power engineering, construction industry, mechanical engineering and other industries, the most promising is the development and improvement of repair technologies aimed at restoration of the structure’s bearing capacity [4,5,6]. According to the results of studies [7], the rational method for repair of the most technological and operational defects in load–bearing structures is the use of repair patches. This repair method consists in layer-by-layer removal of the damaged area and further restoration of structural integrity by using a patch [8]. The efficiency of this method can be increased when the patches are made of modern polymeric composite materials (PCM) [9,10]. PCM have high physical–mechanical and chemical properties, allowing them to reduce the labour intensity of repair significantly [11,12]. The features of their application are listed below [13,14]:

- repairs with the use of such materials do not require sophisticated equipment and high level of training of workers;
- there is no need to disassemble the components and units in the performance of repairs with the composites;
- most often, the use of PCM allows exclusion of welding, depositing or soldering, and also it is possible to perform repairs of the products and assemblies which cannot be restored with the use of the other known methods, as well as repairs in hazardous conditions;
- the use of PCM allows restoring the parts without the need for complex technological processes of coating with materials and subsequent processing.

The simplicity of preliminary surface preparation and technological process of bonding of the composite patch and possibility of performance of repairs in the field make this method effective both from the mechanical and economic points of view [15].

Nevertheless, despite numerous advantages, the practical application of this method, as a rule, is limited to the repair of critical composite structures [16,17]. Moreover, even in these cases it is not always possible to obtain the optimal results, since the repaired structure often has the overstated bearing capacity [18]. Therefore, improper choice of the patch parameters results in the unjustified increase in the mass of the structure and cost of its further operation [19,20,21]. The reason is the lack of reliable scientifically grounded techniques and algorithms for the repair process development, which take into account the influence of the geometry of the patch, the process of its moulding-on and the conditions for the performance of repair works on the bearing capacity of the repaired structure. Development of such techniques will allow providing the regulated restoration of bearing capacity of a damaged structure with the minimal impact on its characteristics (stiffness, weight, bearing capacity, etc.) and at the same time reducing the costs and time for repairs by expansion of the scope of its application.

**Figure 1:**
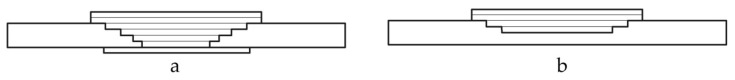
Pattern of installation of the composite repair patch for through defect (**a**) and partially through defect (**b**).

Currently, one of the most applicable patterns of installation of a composite patch is the removal of material with the simultaneous formation of steps (Figure 1). This pattern of installation of the composite repair patch is used for the repair of a wide range of operational defects in structures: dents and delaminations in composite aggregates; surface defects of metal aggregates caused by corrosion; cracks and perforation damages in aggregates of any type [22,23].

Repair of structures by moulding-on (bonding) of the composite patch leads to formation of an adhesive layer on the panel–patch interface. It is the layer of low stiffness capable of receiving significant transverse shear deformations during operation of the repaired structure. Therefore, the classical theories of layered plates and shells cannot be used here to calculate the stress–strain behaviour [24,25]. At the same time, the existing refined theories, taking into account the transverse shear [26,27] in whatever way, are not intended for modelling of structures of step-variable thickness. One of the aspects of these problems is the development of more reliable methods for determining the stress–strain behaviour near the area of distortion of the stress state, i.e., the area near the patch attachment and stepped variation of thickness of the structure being repaired [28]. This is explained by the fact that for these cases the classical theory does not give satisfactory agreement with practice because of the three-dimensional nature of the stress–strain behaviour [29,30].

The paper [31] offers a modified approach to analytical modelling of the effect of the patch attachment area on the stress–strain behaviour of a weakened composite panel. The traditional method for determination of the total potential energy has been extended by introducing the modified functional. This functional is similar to the total potential energy and can take into account full mechanical coupling of the layers cut in steps. The method for predicting the stress–strain behaviour of composite plates weakened by damage is described in [32]. The method is based on the new pattern for dividing the laminate package, which provides better consistency of displacement near the stepped variation of thickness of the structure in the damage zone. The analytical model for studying the stress–strain behaviour of composite plates with the rectangular zone of damage is developed in [33]. The proposed model allowed investigation of the global, local and mixed response to bending of the composite plates weakened by damage. The paper [34] deals with the effect of interlayer strength, damage area and its depth on the residual strength of the damaged panel. A three-dimensional model of the composite panel damage is developed in [35]. The model takes into account the mechanisms of failure of fibres and matrix under tension and compression, when the damaged panel is subjected to a three-dimensional stress state. The paper [36] develops the mathematical model for determination of the stress–strain behaviour of the panel with asymmetric stepped variation of thickness. The method of direct asymptotic integration of differential equations of the theory of elasticity in three dimensions is used. The formulated boundary value problem is a system of two differential equations of displacements of the second and fourth orders with variable coefficients and corresponding boundary conditions. However, analysis of the formulated boundary value problems showed that their solution was associated with mathematical difficulties. Optimization of the shape of the stepped composite patch, which allows decreasing the bonding area by 33–40% compared to the traditional design of the patch of similar strength, is carried out in [37]. The paper [38] deals with construction of two-dimensional equations for an arbitrary anisotropic shell of step-variable thickness based on minimization of the Lagrange energy functional. In this case, the components of the stress–strain behaviour are approximated by polynomial series on the normal coordinate. Conditions for the consistency of expansions for displacements and minimization of the resulting residuals, as well as boundary conditions, are formulated. The similar approach to simplification of a three-dimensional problem is described in [39]. The method for assessment of the stress–strain behaviour of the damaged panel composite structure is developed. This method allows determining the degree of stress concentration in the area of stepped variation of thickness, and identifying the most dangerous point where the structure failure can start. The deficiency of this study is that the results are obtained for the flat plate only. It does not allow generalization of the results obtained on a panel of arbitrary shape and curvature. 

In connection with the growth of computational capabilities, the classical analytical methods of calculation are replaced by numerical ones, in particular, by the finite element method [40]. The refined finite element model, which allow assessing the strength of the damaged composite panel, is developed in [41]. However, the main disadvantage of the numerical approach is that the finite element model is created for the strictly defined structure and cannot be extended arbitrarily to similar elements. The most adequate assessment of the bearing capacity of composite structures with the peculiar features under study is possible with the use of experimental studies [42]. The papers [43,44] deal with the experimental study of the effect of size and location of damage on the bearing capacity of the composite panel. 

The paper [45] analyses the effect of the length of the repair patch on the repair characteristics using the experimental and numerical approaches. The stress intensity factor and adhesion stresses were determined for the various repair patch sizes. Results of experiments showed that strength of repaired plates decreased significantly with the increase in the length of the repair patch, regardless of the material of the structure. Numerical results demonstrate that the stress intensity factor is growing with increase in the length of the repair patch.

Based on the analysis, currently there is an urgent need to develop the mathematical model for determining the stress–strain behaviour of the reparable composite shell structure of step-variable thickness at the strength, temperature and shrinkage loading.

## 2. Materials and Methods

The problem of determination of the stress–strain behaviour of a shell structure is solved in the linear formulation by the methods of the theory of elasticity using the basic dependencies of the composite mechanics. To calculate the stress–strain behaviour, the layer-by-layer theory is chosen. Stepped variation of thickness was modelled by introducing special functions for the patch and notch. The problem was solved using the energy technique. The reliability of the developed mathematical model is confirmed by comparison with the results of experimental and numerical studies. The results of experimental studies presented in the paper were obtained in laboratory conditions using standard equipment, instruments and tools. AeroGlass–163 (Havel Composites CZ s.r.o, Svésedlice, Czech Republic) glass cloth and hot–cured binder based on Epikure (RPP, London, UK) epoxy resin and Epikote (HEXION, Vondelingenplaat, The Netherlands) curing agent were used as the materials for the sample preparation. In the process of experimental studies, the method of double-exposure holographic interferometry was used with the registration of holograms in counter–propagating beams. Numerical studies were carried out in a finite element analysis software package. We used two types of finite elements: 4–node multilayer four–angled shell element with flexural and membrane properties for spatial analysis: ShellL type, six degrees of freedom (three translations and three rotations were considered per node) and an 8–node multilayer 3D finite element SolidL type (three translational degrees of freedom are considered for each node).

## 3. Theoretical Background

**Figure 2:**
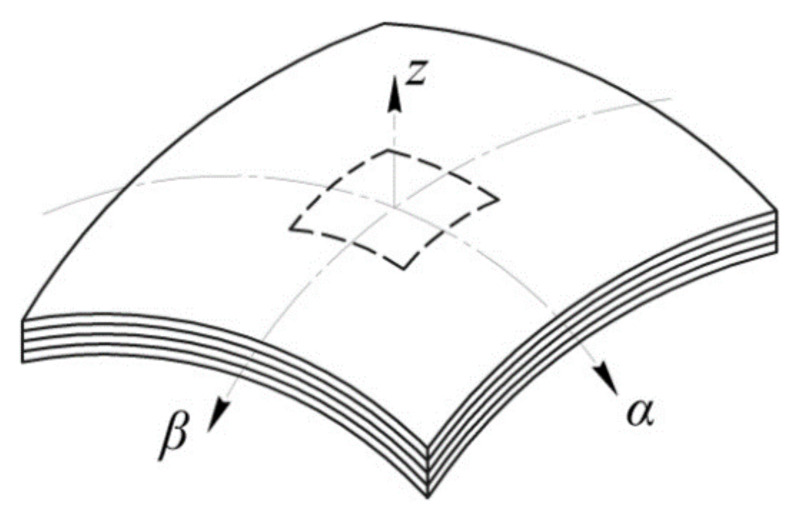
Geometric parameters of the panel of step-variable thickness.

Here we consider the general case of geometry of the panel structure element–anisotropic shell of double curvature (Figure 2). The shell is formed by a set of PCM layers differently oriented in the plane and having arbitrary physical and mechanical characteristics. The projection of the shell onto the plane passing through the vertices of its contour is a rectangle. The shell under study features stepped variation of thickness, representing a patch or notch of arbitrary shape in the plan, which can be described by the algebraic or parametric curve.

We assume that most often the notch or patch have a rectangular shape, as it is the simplest shape widely used in practice [33,39]. Figure 1 shows the boundary at which the shell thickness variates stepwise by an amount corresponding to thickness of the patch or depth of the notch, by a dashed line (Figure 2).

We introduce the orthogonal coordinate system (*α, β, z*) so that the lower surface of the shell is referred to the curvilinear coordinates *α* and *β*, and the rectilinear coordinate axis *z* is directed upward along the normal to this surface. Let the shell consist of the layers being anisotropic in the chosen curvilinear coordinate system. For each of the layers, physical and mechanical characteristics of the material and the angle of orientation of the fibres with regard to axes of the common coordinate system are known (*α, β, z*).

The following assumptions are made:

- there is no reduction across the thickness of the PCM package, therefore *ε_z_* = 0 and *w_i_* = *W*(*α, β*);
- transverse normal stresses *σ_z_* are negligible compared to the principal stresses and they are not taken into account in the calculations, i.e., *σ_z_* = 0;
- within each individual layer, the hypothesis of a straight line is fulfilled, while the condition of continuity of displacement and stresses is satisfied at the interface between layers.

In the general case, the shell under study can be fixed in an arbitrary way and loaded with any external force factors. According to a number of papers [24,25], in the studies of real structures the most common patterns for the fixation of panels can be quite accurately described by the boundary conditions below

(1) $$\alpha =\pm \frac{a}{2};w=0,\;\;v=0,\;\theta_{\beta }=0,\;\;N_{\alpha }=0,\;\;M_{\alpha }=0,\;\beta =\pm \frac{b}{2};w=0,\;\;v=0,\;\theta_{\alpha }=0,\;\;N_{\beta }=0,\;\;M_{\beta }=0.$$

Boundary conditions (1) correspond to free bearing of the panel edges and assume the absence of bending of the contour points and their displacement along the edge. The possibility of free displacement of the contour points across the edge is based on the possibility of deformation of the basic structure from the plane.

As external force factors, we assume the action of a transverse distributed and concentrated load, as well as the impact of temperature and shrinkage deformations. 

**Figure 3:**
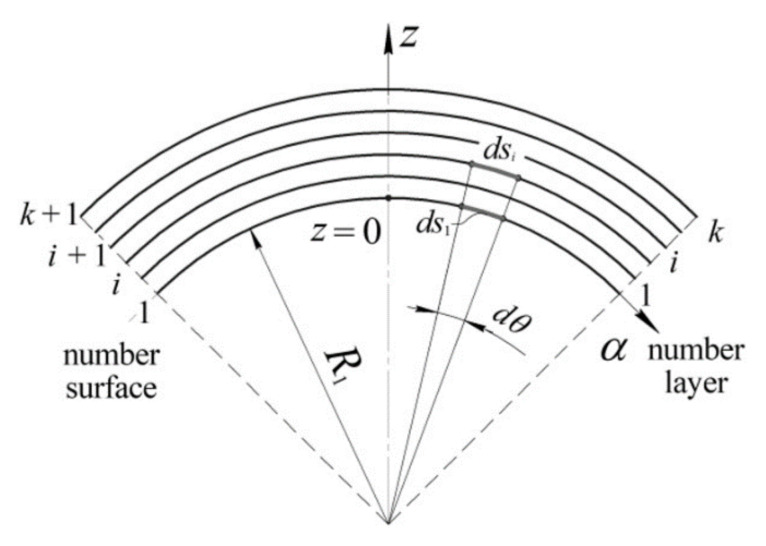
Shell element.

For the shell element of double curvature (Figure 3) with constant radii *R*_1_ and *R*_2_, referring to the chosen coordinate system, Lamé coefficients *A*_1_ and *A*_2_ can be determined based on the following:

(2) $$ds_{1}=Rd\theta =1d\alpha ,\;ds_{i}=\left(R+z_{i}\right)d\theta =\left(1+\frac{z_{i}}{R}\right)d\alpha \;$$

According to Equation (2), Lamé coefficients of an arbitrary surface of the considered shell are determined by the formulas:

(3) $$A_{1}=1+\frac{z}{R_{1}},\;A_{2}=1+\frac{z}{R_{2}},\;A_{3}=1.$$

**Figure 4:**
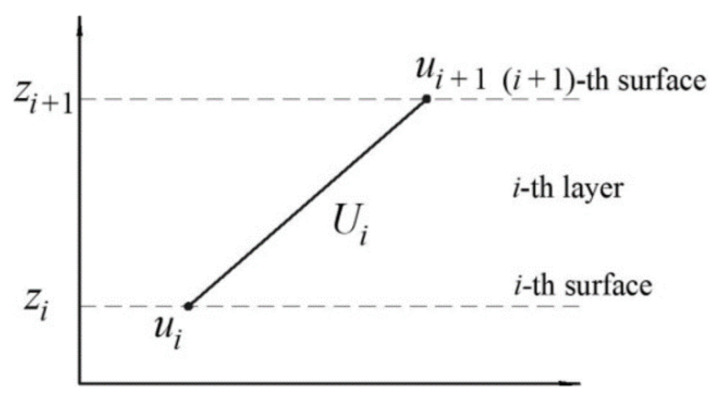
Approximation of displacements within the i–th layer.

To reduce the three–dimensional problem of elasticity to the two–dimensional one, displacements *u_i_*, *v_i_* and *w_i_* are set for the certain point of the *i*–th surface of the shell as a function of variables *α* and *β* in order to satisfy the specified boundary conditions on the edges determined by dependencies (1). The *i*–th surface of the panel is understood as the mating surface of the (*i*−1)–th and *i*–th layers. In accordance with the assumption about fulfilment of the hypothesis of a straight line within the layer, the distribution of displacements *U_i_* and *V_i_* over the thickness of the *i*–th layer follows the linear law (Figure 4).

According to Figure 4 the displacement *U_i_* is determined as follows:

(4) $$U_{i}=u_{i}+\frac{1}{\delta_{i}}\left(u_{i+1}-u_{i}\right)\left(z-z_{i}\right).$$

Similarly, we define the displacements *V_i_*:

(5) $$V_{i}=v_{i}+\frac{1}{\delta_{i}}\left(v_{i+1}-v_{i}\right)\left(z-z_{i}\right).$$

The displacement *W* is constant for all layers of the plate, owing to the assumption about no reduction.

In the framework of the linear theory, deformations arising in the shell are associated with displacements by geometric relations (6), obtained by substituting the Lamé coefficients (3) into the known equations [24,25,27]:

(6) $$\varepsilon_{\alpha }^{i}=\frac{R_{1}}{z+R_{1}}\frac{\partial U_{i}}{\partial \alpha }+\frac{1}{z+R_{1}}W,\;\varepsilon_{\beta }^{i}=\frac{R_{2}}{z+R_{2}}\frac{\partial V_{i}}{\partial \beta }+\frac{1}{z+R_{2}}W,\;\gamma_{\alpha \beta }^{i}=\frac{R_{2}}{z+R_{2}}\frac{\partial U_{i}}{\partial \beta }+\frac{R_{1}}{z+R_{1}}\frac{\partial V_{i}}{\partial \alpha },\;\gamma_{\alpha z}^{i}=-\frac{U_{i}}{z+R_{1}}+\frac{\partial U_{i}}{\partial z}+\frac{R_{1}}{z+R_{1}}\frac{\partial W}{\partial \alpha },\;\gamma_{z\beta }^{i}=-\frac{V_{i}}{z+R_{2}}+\frac{\partial V_{i}}{\partial z}+\frac{R_{2}}{z+R_{2}}\frac{\partial W}{\partial \beta }.$$

Since, in the general case, material of an individual layer of the PCM package at each point has only one plane of elastic symmetry parallel to the median surface of the shell, and stresses *σ_z_* are negligible, the solution of the Hooke’s law with respect to stresses is written as:

(7) $$\sigma_{\alpha }^{i}=b_{11}^{i}\varepsilon_{\alpha }^{i}+b_{12}^{i}\varepsilon_{\beta }^{i}+b_{13}^{i}\gamma_{\alpha \beta }^{i},\;\sigma_{\beta }^{i}=b_{12}^{i}\varepsilon_{\alpha }^{i}+b_{22}^{i}\varepsilon_{\beta }^{i}+b_{23}^{i}\gamma_{\alpha \beta }^{i},\;\sigma_{\alpha \beta }^{i}=b_{13}^{i}\varepsilon_{\alpha }^{i}+b_{23}^{i}\varepsilon_{\beta }^{i}+b_{33}^{i}\gamma_{\alpha \beta }^{i},\;\tau_{\alpha z}^{i}=b_{44}^{i}\tau_{\alpha z}^{i}+b_{45}^{i}\gamma_{\beta z}^{i},\;\tau_{\beta z}^{i}=b_{45}^{i}\tau_{\alpha z}^{i}+b_{55}^{i}\gamma_{\beta z}^{i}.$$

Stiffness matrix coefficients in the equations (7) are calculated using the known formulas [24,25,27].

The complex shape of bending of the panel with step–variable thickness is the reason for the choice of the energy technique to solve the problem [39]. Total deformation energy of the elastic system is written as:

(8) E=P+A,

where *P*—potential energy of deformation; *A*—total work of external forces applied to the panel.

Potential energy of deformation of the *i*–th layer of the panel is determined as:

(9) $$P_{i}=\frac{1}{2}\iint_{S}\int_{z_{i}}^{z_{i+1}}\left\{\sigma^{i}\varepsilon^{i}\right\}A_{1}A_{2}\;d\alpha d\beta dz,$$

where *z_i_* and *z*_(*i*+1)_—coordinates of the surfaces confining the *i*–th layer (Figure 3).

In this case, the potential energy of deformation of the entire panel is equal to the sum of the potential energies of deformation of its layers:

(10) $$P=\sum_{i=1}^{k}P_{i}.$$

If the panel is subjected to action of the uniformly distributed transverse pressure, we define the work of external forces as:

(11) $$A\left(p\right)=-\iint_{S}pWH_{1}H_{2}\;d\alpha d\beta ,$$

where *p*—intensity of the uniformly distributed transverse pressure; and *S*—area of the panel surface section to which the external load is applied. 

**Figure 5:**
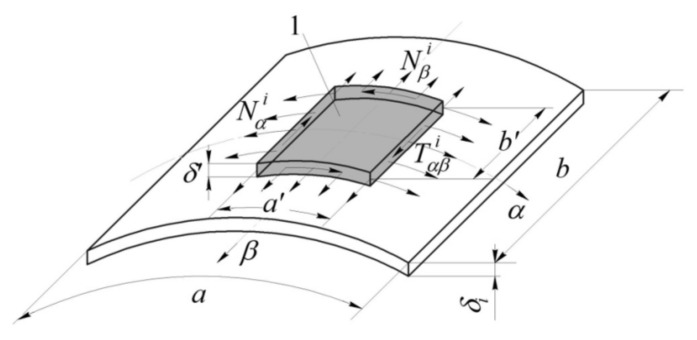
Approximation of displacements within the *i*–th layer: 1—area of temperature changes in the *i*-th layer.

To determine the stress–strain behaviour in the panel as a result of thermal or shrinkage loading, we additionally calculate the work of internal forces $N_{\alpha }^{i}$, $N_{\beta }^{i}$ and $T_{\alpha \beta }^{i}$ (Figure 5):

(12) $$A\left(N_{\alpha }\right)={\left[\int_{\beta =-\frac{b^{\prime }}{2}}^{\beta =\frac{b^{\prime }}{2}}N_{\alpha }^{i}U_{i}A_{2}d\beta \right]}_{\alpha =-\frac{a^{\prime }}{2}}^{\alpha =\frac{a^{\prime }}{2}};\;\;A\left(N_{\beta }\right)={\left[\int_{\alpha =-\frac{a^{\prime }}{2}}^{\alpha =\frac{a^{\prime }}{2}}N_{\beta }^{i}V_{i}A_{1}d\alpha \right]}_{\beta =-\frac{b^{\prime }}{2}}^{\beta =\frac{b^{\prime }}{2}},\;A\left(T_{\alpha \beta }\right)={\left[\int_{\beta =-\frac{b^{\prime }}{2}}^{\beta =\frac{b^{\prime }}{2}}T_{\alpha \beta }^{i}V_{i}A_{2}d\beta \right]}_{\alpha =-\frac{a^{\prime }}{2}}^{\alpha =\frac{a^{\prime }}{2}}+{\left[\int_{\alpha =-\frac{a^{\prime }}{2}}^{\alpha =\frac{a^{\prime }}{2}}T_{\alpha \beta }^{i}U_{i}A_{1}d\alpha \right]}_{\beta =-\frac{b^{\prime }}{2}}^{\beta =\frac{b^{\prime }}{2}},$$

where $N_{\alpha }^{i}=\int_{z_{i}}^{z_{i+1}}\sigma_{\alpha T}^{i}dz;\;N_{\beta }^{i}=\int_{z_{i}}^{z_{i+1}}\sigma_{\beta T}^{i}dz$; $T_{\alpha \beta }^{i}=\int_{z_{i}}^{z_{i+1}}\tau_{\alpha \beta T}^{i}dz$—resultant values of total temperature and shrinkage stresses $\sigma_{\alpha T}^{i}$, $\sigma_{\beta T}^{i}$ and $\tau_{\alpha \beta T}^{i}$, arising in the *i*–th layer of the shell.

The total temperature and shrinkage deformations $\varepsilon_{\alpha T}^{i}$, $\varepsilon_{\beta T}^{i}$ and $\gamma_{\alpha \beta T}^{i}$ shall be written as:

(13) $$\varepsilon_{\alpha T}^{i}{}^{*}=\alpha_{\alpha }^{i}\Delta T-\xi_{\alpha }^{i},\;\varepsilon_{\beta T}^{i}{}^{*}=\alpha_{\beta }^{i}\Delta T-\xi_{\beta }^{i},\;\gamma_{\alpha \beta T}^{i}{}^{*}=\alpha_{\alpha \beta }^{i}\Delta T-\xi_{\alpha \beta }^{i}.$$

Total stresses $\sigma_{\alpha T}^{i}$, $\sigma_{\beta T}^{i}$ and $\tau_{\alpha \beta T}^{i}$ are determined by substitution of the total temperature and shrinkage deformations $\varepsilon_{\alpha T}^{i}$, $\varepsilon_{\beta T}^{i}$ and $\gamma_{\alpha \beta T}^{i}$ (13) instead of the general deformations $\varepsilon_{\alpha }^{i}$, $\varepsilon_{\beta }^{i}$ and $\gamma_{\alpha \beta }^{i}$, accordingly, into the formulas (7).

For the determination of stress–strain behaviour of the shell of step-variable thickness, we use the Ritz–Timoshenko method [25,27]. This method allows us to obtain the approximate solution in displacements based on the Lagrange variational principle. In accordance with the chosen method, for the panel of arbitrary shape the displacements of its *i*–th surface *u_i_*, *v_i_*, *W* are set in the general form below:

(14) $$u_{i}=\left(\frac{\beta^{2}}{b^{2}}-1\right)\sum_{m=0}^{N}\sum_{n=0}^{m}{\bar{u}}_{m,n,i}{\left(\frac{\alpha }{a}\right)}^{m-n}{\left(\frac{\beta }{b}\right)}^{n},\;v_{i}=\left(\frac{\alpha^{2}}{a^{2}}-1\right)\sum_{m=0}^{N}\sum_{n=0}^{m}{\bar{v}}_{m,n,i}{\left(\frac{\alpha }{a}\right)}^{m-n}{\left(\frac{\beta }{b}\right)}^{n},\;W=\left(\frac{\alpha^{2}}{a^{2}}-1\right)\left(\frac{\beta^{2}}{b^{2}}-1\right)\sum_{m=0}^{N}\sum_{n=0}^{m}{\bar{w}}_{m,n}{\left(\frac{\alpha }{a}\right)}^{m-n}{\left(\frac{\beta }{b}\right)}^{n},$$

where *N*—degree of the polynomial determining the panel displacement; and ${\bar{u}}_{m,n,i}$; ${\bar{v}}_{m,n,i}$ and ${\bar{w}}_{m,n}$—unknown coefficients to be determined. In accordance with our results, the values presented below were obtained at *N* = 12 for the case of asymmetric location of the notch and *N* = 6 for the symmetric problem. The search for displacements in the form of (14) allows us to obtain a solution for problems with the loading and/or location of the notch/patch, which is asymmetric with regard to axes (*α*, *β*). 

Equating to zero the first variation of the total energy of the system *δE*, we obtain a complete system of equations describing the equilibrium state of the deformed shell to determine the unknown coefficients of the series (14):

(15) $$\frac{\delta E}{{\bar{u}}_{m,n,i}}=0,\;\;\frac{\delta E}{{\bar{v}}_{m,n,i}}=0,\;\;\frac{\delta E}{{\bar{w}}_{m,n}},$$

Since the total energy is the quadratic function of displacements, Equation (15) represents a complete system of the linear algebraic equations for the coefficients being determined. After finding the coefficients ${\bar{u}}_{m,n,i}$; ${\bar{v}}_{m,n,i}$ and ${\bar{w}}_{m,n}$ by solving of this system, authors can determine the displacements and then deformations and stresses with the use of formulas (14), that is, we obtain a complete solution to the problem. The solution of system (15) allows determining the displacements, deformations and stresses at an arbitrary point of the shell.

Modelling of the stepped variation of the panel thickness is performed by introduction of the additional functions $H_{i}^{\prime }$ and $H_{i}^{\prime \prime }$ for the patch and notch, accordingly:

(16) $$H_{i}^{\prime }=Hev\left(\alpha -\alpha_{1}^{i}\right)Hev\left(\alpha -\alpha_{2}^{i}\right)Hev\left(\beta -\beta_{1}^{i}\right)Hev\left(\beta -\beta_{2}^{i}\right),\;H_{i}^{\prime \prime }=1-Hev\left(\alpha -\alpha_{1}^{i}\right)Hev\left(\alpha -\alpha_{2}^{i}\right)Hev\left(\beta -\beta_{1}^{i}\right)Hev\left(\beta -\beta_{2}^{i}\right),$$

where *Hev*—the Heaviside function, $\alpha_{1}^{i},\;\;\alpha_{2}^{i}$—coordinates of the right and left boundaries along the axis α of the stepped variation of the plate thickness in the *i*–th layer; $\beta_{1}^{i},\;\;\beta_{2}^{i}$—coordinates of the right and left boundaries along the axis α of the stepped variation of the panel thickness in the *i*–th layer.

Using the functions $H_{i}^{\prime }$ and $H_{i}^{\prime \prime }$, modeling of a panel with any geometric parameters in the plan and across the thickness and combined physical and mechanical characteristics is possible by setting the stiffness matrix coefficients for layers of the patch or notch ${\bar{b}}_{ij}\;$ as follows:

(17) $${\bar{b}}_{ij}=H_{i}^{\prime }\left(H_{i}^{\prime \prime }\right)b_{ij},$$

where $b_{ij}$—stiffness matrix coefficients of the intact layer.

The use of the above approach allows studying the distribution of stresses over the thickness of the panel, as well as estimating their concentration in the area of sharp change in stiffness.

**Figure 6:**
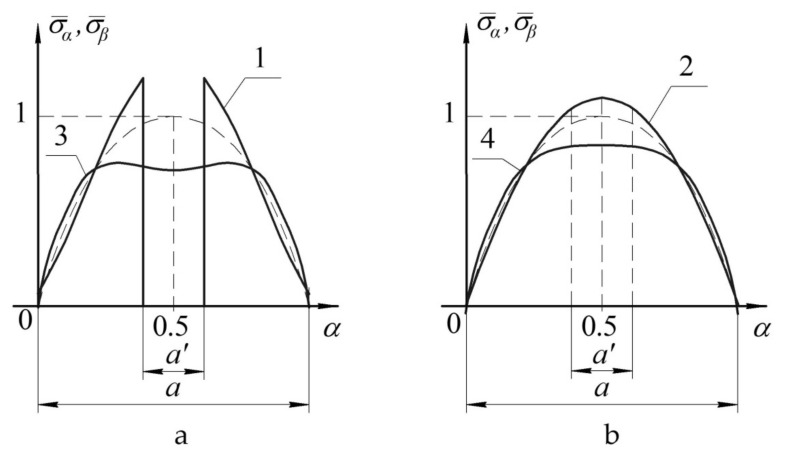
Distribution of normal stresses in the notched panel (1,2) and panel with a patch (3,4) on upper (**a**) and lower (**b**) surfaces.

**Figure 7:**
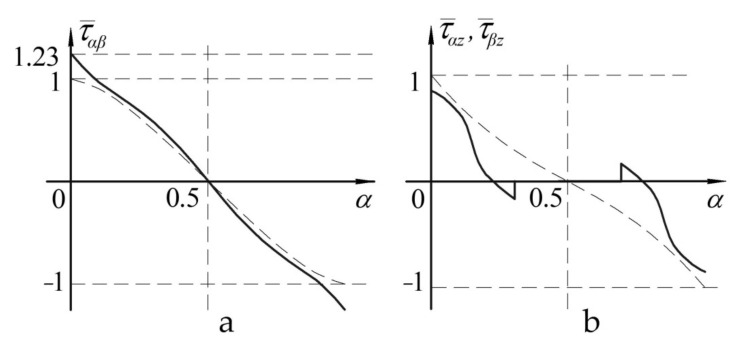
Distribution of tangential stresses on the upper surface of the panel (**a**) and transverse shear stresses in the layer with a notch (**b**).

The graphs shown in Figure 6 reflect the distribution of normal stresses on upper (Figure 6a) and lower (Figure 6b) surfaces of notched panel (lines 1,2) and panel with a patch (lines 3,4). The graphs shown in Figure 7 reflect the distribution of tangential stresses on the upper surface of the panel (Figure 7a) and transverse shear stresses in the layer with a notch (Figure 7b).

The nature of changes in the stress distribution is represented as the dependence diagrams of distribution of the dimensionless normal and tangential stresses in the panel under study, determined as follows: $\bar{\sigma }=\sigma /\sigma_{0}^{max}$, $\bar{\tau }=\tau /\tau_{0}^{max}$, where *σ,* *τ*—stresses acting in the panel; and $\sigma_{0}^{max}$, $\tau_{0}^{max}$—maximum stresses in the panel of the uniform thickness.

The graphs shown in Figure 6 and Figure 7 allowed drawing the conclusions below:

- in the case of the notched panel, maximum increase in stresses will occur in the weakened layer, and it is from this layer the failure of the structure will start;
- in the case of panel with a patch, the panel surface opposite to the reinforcement is the most loaded (i.e., susceptible to failure) surface.

In order to assess the influence of the patch shape, we conducted the numerical study of the stress–strain behaviour arising under the action of the uniform transverse load in the rectangular panel. Modelled panel is made of unidirectional carbon fibre composite with the physical and mechanical characteristics below: elastic modulus along the fibres *E*_1_ = 100 GPa; elastic modulus across the fibres *E*_2_ = 10 GPa; shear modules *G*_12_ = 6 GPa; *G*_23_ = *G*_13_ = 7 GPa; Poisson’s ratio *μ*_12_ = 0.35; coefficient of linear thermal expansion along the fibres *α*_1_ = 0 and across the fibres *α*_2_ = 3 × 10^−5^; tensile strength along the fibres *F*_1*t*_ = 900 MPa; compressive strength along the fibres *F*_1*c*_ = 700 MPa; tensile strength across the fibres *F*_2*t*_ = 50 MPa; compressive strength across the fibres *F*_2*c*_ = 120 MPa; shear strength *F*_12_ = 75 MPa. The panel is weakened by a central circular hole. A patch made of similar material had the uniform thickness and area in all calculations. 

To assess the degree of loading of panel $\;K_{0}$ and patch $K_{p}$, the Von Mises–Hill energy criterion of strength has been chosen [24,27]:

(18) $$K=\sqrt{\frac{\sigma_{\alpha }^{2}}{F_{1}^{2}}-\frac{\sigma_{\alpha }\sigma_{\beta }}{F_{1}F_{2}}+\frac{\sigma_{\beta }^{2}}{F_{2}^{2}}+\frac{\tau_{\alpha \beta }^{2}}{F_{12}^{2}}+\frac{\tau_{\alpha z}^{2}}{F_{13}^{2}}+\frac{\tau_{\beta z}^{2}}{F_{23}^{2}}}\;,$$

where $\sigma_{\alpha }$, $\sigma_{\beta }$, $\tau_{\alpha \beta }$, $\tau_{\alpha z}$, $\tau_{\beta z}$—effective normal and tangential stresses in the panel or patch, $F_{1}$, $F_{2}$, $F_{12}$, $F_{13}$, $F_{23}$—ultimate strength of PCM used for the panel or patch.

In order to assess the degree of reduction of the strength of adhesive interlayer between the panel and the patch to prevent it from peeling off in the process of operation, we used the loading factor equal to:

(19) $$K_{ad}=\frac{\tau_{ad}}{F_{ad}}\;,$$

where $\tau_{ad}$—transverse shear stresses in the adhesive interlayer; and $F_{ad}=50$ MPa—adopted ultimate strength of the adhesive interlayer. 

**Figure 8:**
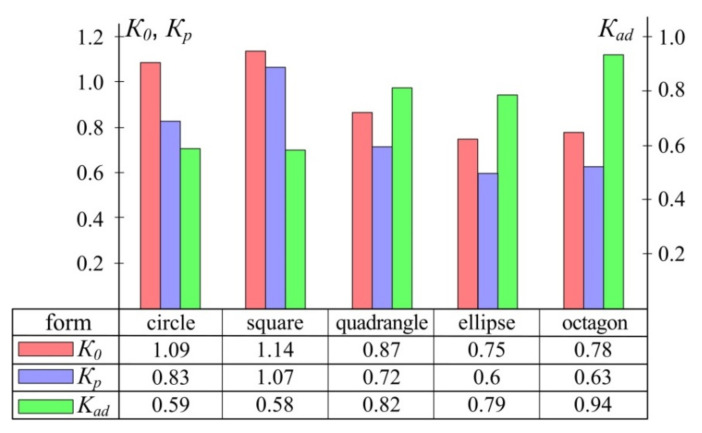
Effect of the patch shape on the degree of loading of the repaired panel ($K_{0}$), patch ($K_{p}$) and adhesive interlayer between them ($K_{ad}$).

**Figure 9:**
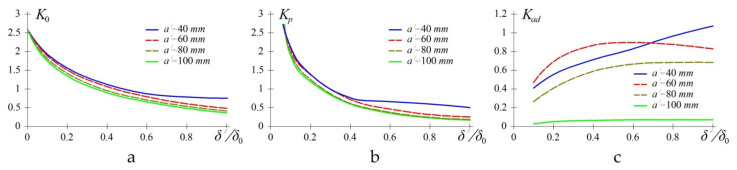
Effect of thickness $\delta^{\prime }$ and size of patch $a^{\prime }$ on the loading factor of the repaired panel (**a**) of thickness $\delta_{0}$; loading factor of the patch (**b**) and adhesive interlayer (**c**).

The results are shown in Figure 8. Results of assessment of the effect of dimensions of the composite patch on the degree of loading of the repaired panel, the patch itself and adhesive interlayer between them are shown in Figure 9. To repair through the defect, a rectangular patch of the uniform cross-section with the varying transverse dimensions and thickness was used.

When analysing the results, we can draw the following conclusions. The change in the patch thickness $\delta^{\prime }$ has the maximum effect on the loading factor of the repaired panel $K_{0}$ and patch $K_{p}$. For the considered example, a two-fold increase of $\delta^{\prime }$ leads to the reduction of loading of the repaired panel by 18–45% and the patch itself by 34–60%, depending on the patch size.

Loading factors of the repaired panel $K_{0}$ and patch $K_{p}$ have low sensitivity to the patch size $a^{\prime }$. An increase of its dimensions by half reduces loading of the panel by 9.6–15% and a patch by 9.15–16.5%, depending on the patch thickness.

**Figure 10:**
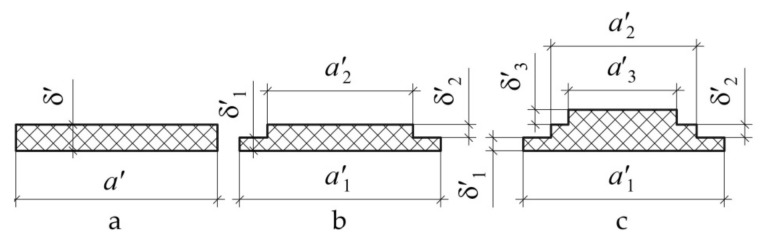
Geometrical dimensions of the cross section of repair patch of uniform thickness (**a**) and variable thickness with two (**b**) and three (**c**) steps.

Table 1 represents the results of numerical studies of the effect of the patch cross-section shape (Figure 10a).

The studies were carried out for the following input data. Dimensions of the repaired panel: 100 × 200 × 2 mm. We used carbon fibre composite with the above physical and mechanical characteristics. The defect was a through hole of 20 mm in diameter located in the centre of the panel. The repair patch was rectangular in shape with the dimensions ratio of 1:2 of carbon fibre of $\delta^{\prime }=0.2$ mm thick. Adhesive: modulus of elasticity *E = 5* GPa, Poisson’s ratio *μ* = 0.3, ultimate strength $F_{ad}=35$ MPa.

Table 2 represents the results of numerical studies of the effect of the patch cross-section shape (Figure 10b). 

Table 2 represents the results of numerical studies of the effect of the patch cross-section shape (Figure 10c).

Based on the results (Table 3), the rational geometric parameters of the repair patches were determined which satisfied the criteria of operability of the repaired structure ($K_{0}\to 1,K_{p}\to 1,\;K_{ad}\to 1\;$) and the condition for the minimum mass.

The studies were carried out for the following input data. Dimensions of the repaired panel: 100 × 200 × 2 mm. We used carbon fibre composite with the above physical and mechanical characteristics. The defect was a through hole of 20 mm in diameter located in the centre of the panel. The repair patch was rectangular in shape with the dimensions ratio of 1:2 of carbon fibre of $\delta^{\prime }=0.2$ mm thick. Adhesive: modulus of elasticity *E = 5* GPa, Poisson’s ratio *μ* = 0.3, ultimate strength $F_{ad}=35$ MPa. Table 4 represents rational geometric parameters.

After analysis of the results, we can state the following:

- The main criterion for selection of parameters of the patch of uniform thickness is the restoration of strength of the panel under repair when the condition of operability of the adhesive layer is satisfied. In this case, the repair patch remains significantly underloaded (in the considered example, by 35%).
- Transition to a patch of variable thickness considerably reduces the stresses in the adhesive layer (in the considered example, by 20–25%). It allows reduction of the overall dimensions of the repair patch.

## 4. Experimental Research

To verify the results of theoretical calculations according to the proposed mathematical model, the deformed state of the notched panel under action of the uniformly distributed transverse load was experimentally investigated. In order to simplify the sample preparation, experiments were carried out on flat panels [39]. Because of non–uniformity of the deformation field caused by stepped variation of the thickness of samples under study, method of strain measurement was not effective [46]. At the same time, a number of optical methods allow obtaining the displacement field in the structure under study and comparing it with analytical results [7]. In the process of experimental research, we applied the method of double-exposure holographic interferometry with the registration of holograms in counter-propagating beams. This method allowed us to compare two deformed states of the object under study, where each of them was characterized by the applied load (in this experiment it was the uniformly distributed pressure p and displacement w caused by this load). The result of the hologram reconstruction is an interference pattern, which carries information about displacements of points on the object surface caused by an increment in the external load. Interpretation of the interference pattern consists in plotting of graphs of the normal displacements of the surface of the object under study on the section of interest.

**Figure 11:**
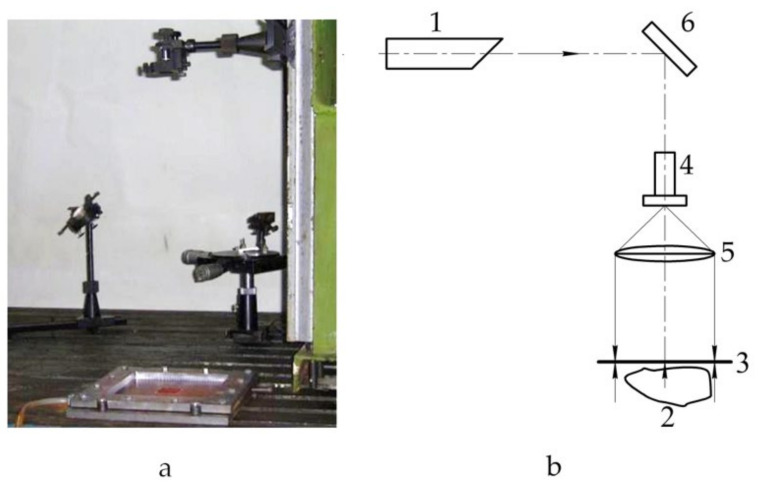
General view (**a**) and schematic diagram (**b**) of the experimental unit: 1—laser; 2—object of research; 3—recording plate; 4—microlens; 5—collimating lens; 6—mirror.

The general view of the experimental unit for hologram recording is shown in Figure 11a, the schematic diagram—in Figure 11b.

For sample testing, we used a tool made in the form of metallic plate with a square cavity for pressurization and rigid frame. The test sample was placed between these two plates on the thin layer of sealant and clamped along the contour with 16 bolts. Therefore, with the securing of tightness the boundary conditions matching as closely as possible the modelled ones were created. Since the monolithic composite panels were characterized by low flexural stiffness, the uniform external pressure on the sample was created by pumping the liquid to the tool cavity and controlled with high accuracy according to the difference in the liquid column.

AeroGlass–163 glass cloth (of German origin) and hot-cured binder based on Epikur epoxy resin and Epikot curing agent were used as the materials for the sample preparation with the physical and mechanical characteristics below: elastic modulus along the fibres *E*_1_ = 18.2 GPa; elastic modulus across the fibres *E*_2_ = 18.1 GPa; shear modules *G*_12_ = 9.5 GPa; Poisson’s ratio *μ*_12_ = 0.13.

**Figure 12:**
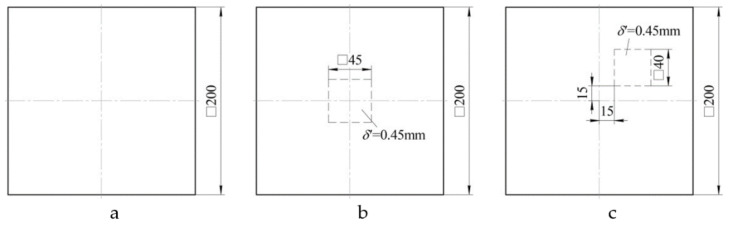
Configuration of three flat plates: of uniform thickness (**a**); with a notch located in the centre (**b**); with a notch located off centre (**c**).

Three flat plates were made of *δ*_0_ = 1.8 mm thick and layering pattern [0°,90°] with the configuration as below:

- uniform thickness (Figure 12a);
- panel with a notch located in the centre (Figure 12b);
- panel with a notch located off centre (Figure 12c).

**Figure 13:**
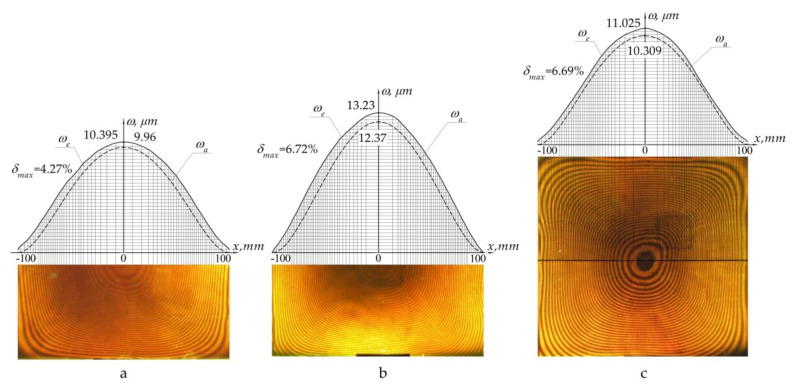
Deflection under the action of distributed pressure *p* of intact panel (**a**); panel with a notch in the centre (**b**); panel with off–centre notch (**c**): *ω_e_*—experimental distribution; *ω_a_*—theoretical (analytical) distribution; *δ_max_*—maximum relative error.

For each type of sample, holographic interferograms were obtained and graphs of normal displacements along the central section of the plate were plotted: panel of uniform thickness (Figure 13a), panel with a notch located in the centre (Figure 13b), panel with a notch located off centre (Figure 13c).

After analysis of the data obtained, the following conclusions can be drawn:

- the displacement field obtained experimentally corresponded to that calculated theoretically;
- the maximum error of numerical values did not exceed 7%, demonstrating good convergence of the results.

## 5. Numerical Implementation

Currently, the high safety and reliability of structures is ensured by conducting a number of full-scale tests [43,44]. Nevertheless, the wide opportunities offered by and high accuracy of calculations in modern finite element software systems allow a reduction the number of experiments to the required minimum and ensuring the lower material costs and time for development of a structure [40,42].

**Figure 14:**
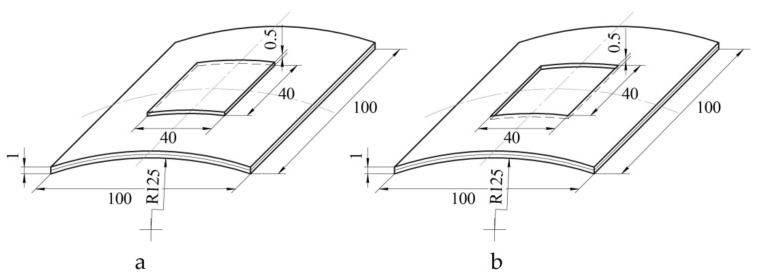
Geometric parameters of panels with a stepped variation of thickness in the form of a notch (**a**) and patch (**b**).

The results obtained demonstrated the good convergence of the deflection fields obtained experimentally and theoretically. However, the limited number of prototypes did not allow a final conclusion about the reliability of the developed mathematical model. In order to obtain the additional data, study of the panel of single curvature with the layering pattern [0°;90°] and stepped variation of thickness in the form of a notch (Figure 14a) and patch (Figure 14b) was conducted with the use of finite element modelling.

The modelled panel is made of unidirectional carbon fibre composite with the physical and mechanical characteristics below: elastic modulus along the fibres *E*_1_ = 100 GPa; elastic modulus across the fibres *E*_2_ = 10 GPa; shear modules *G*_12_ = 6 GPa; *G*_23_ = *G*_13_ = 7 GPa; Poisson’s ratio *μ*_12_ = 0.35; coefficient of linear thermal expansion along the fibres *α*_1_ = 0 and across the fibres *α*_2_ = 3 × 10^−5^; tensile strength along the fibres *F*_1*t*_ = 900 MPa; compressive strength along the fibres *F*_1*c*_ = 700 MPa; tensile strength across the fibres *F*_2*t*_ = 50 MPa; compressive strength across the fibres *F*_2*c*_ = 120 MPa; shear strength *F*_12_ = 75 MPa.

The uniformly distributed lateral load and temperature, acting independently of each other, were considered as external force factors.

We used for calculations one of the software complexes of finite element analysis with capabilities that allowed us to solve a wide range of problems [47]. Construction of the effective finite element model, which allows obtaining, as a result of calculations, the adequate and accurate information about the stress–strain behaviour of the object under study, is largely determined by the correct choice of finite elements. Taking into account the peculiarities of geometry and composition of the structure under study, two types of finite elements were chosen: four-node multilayer four-angled shell element with flexural and membrane properties for spatial analysis (ShellL type, six degrees of freedom (three translations and three rotations were considered per node) and 8-node multilayer three-dimensional finite element (SolidL type, three translational degrees of freedom were considered for each node)).

Given the fact that the error of the finite element method in the determination of the components of stress–strain behaviour of the structures has the order of 1/*n*^2^ (*n*—multiplicity of division into elements by one coordinate), at panel thickness of 1 mm, the grid size in the regular zone was taken to be equal to 0.25 mm. In the area of notch and patch the half as much grid was used, which allowed more accurate modelling of the stress–strain behaviour in the area of stepped variation of thickness. As a result, finite element models with more than 20,000 elements were obtained. Study of the convergence of the numerical solution showed that with such a number of finite elements in the models the normal and shear stresses vary insignificantly (by a maximum 5%). Analysis of the quality of generated finite element models did not reveal any critical errors. 

The problem of determination of the stress–strain behaviour of the shell structure is solved in the linear formulation. The problem of elastic strain of the shell structure has been considered. We considered the panel consisting of k orthotropic layers. It was assumed that the coordinate surface (*α*, *β*) coincided with the lower surface of the plate, and the coordinate axis *z* was directed upwards. For each of the layers, physical and mechanical characteristics of the material and the angle of orientation of the fibres with regard to axes of the common coordinate system are known (*α*, *β*, *z*). A different number of layers simulated the stepped variation of thickness, namely: one element in the notched panel, two elements in the area of regular thickness, and three in the patch area. The selected grid density provided the required calculation accuracy.

The conditions for the fixation of edges of the panels corresponded to the boundary conditions (1) described earlier. For each constructed finite element model we investigated the influence of the lateral-distributed load *p* = 0.1MPa, acting over the entire surface of the shell and temperature change ∆*T* = 100 °C.

Analysis of the results of theoretical calculations and finite element modelling consisted in comparing the nature of the distribution of displacements in the median plane (Table 5) and stresses on the outer surfaces of the panel (Table 6), as well as their maximum values.

Analysis of the resulting data showed the following:

- maximum difference in normal stresses obtained analytically and by the finite element method for various loads was 7.2–10.7% in the case of modelling with multilayer three-dimensional finite elements and 13.6–18.6% in the case of use of multilayer shell elements;
- during comparison of shear stresses arising under the action of pressure, the difference between theoretical values and results of finite element modelling ranged from 3% to 4%;
- in the course of the study of stress–strain behaviour of panels under the effect of temperature, theoretical values of shear stresses are greatly overstated compared to the numerical results of the model based on multilayer 3D finite elements, and differed on average by 15% from the results of modelling using multilayer shell elements.

## 6. Discussion

For the first time, a mathematical model of the composite shell has been developed which takes into account the non-uniformity of transverse shear deformations and simulates stepped variation of thickness of the PCM package by setting the stiffness of the corresponding layers in the form of special functions.

As distinct from the classical theory of layered shells [24,25], the proposed model allowed simplifying the three-dimensional problem by setting of the displacement field on the interfaces between the layers and their linear interpolation over the plate thickness, taking into account the transverse shear deformations. Unlike the papers [26,27], where the transverse shear was taken into account in whatever way, the proposed model allowed modelling the structure of step-variable thickness. It is important for the determination of the stress–strain behaviour near the areas of distortion of the stress state, i.e., the area near the patch attachment and stepped variation of thickness of the structure being repaired [36]. The results of the work provide generalization of the previously obtained results for a flat panel [39] on composite shells of double curvature. Taking these factors into account allows us to proceed to consideration of a broader class of problems.

The obtained results allow modelling of a shell actually with any geometric parameters and combined physical and mechanical characteristics of the composite by setting the corresponding stiffness matrix coefficients. In contrast to the previous works, it allowed us to determine the degree of stress concentration in the area of stepped variation of thickness, the most dangerous point where the structure failure can start. Moreover, it is also possible to assess the stress–strain behaviour in the panel as a result of temperature or shrinkage loading.

According to the results obtained, in the case of a notched panel, maximum increase in stresses will occur in the weakened layer, and it is from this layer that the failure of the structure will start. In case of panel with a patch, surface of the panel opposite to the reinforcement is the most loaded (i.e., susceptible to failure) surface. This confirms the conclusions of a number of papers, such as [33,34,36,37]. The nature of the change in stresses on the lower surface of the plate, free from sharp variation of thickness, is smoother than on the upper one. These features are to be taken into account when designing composite structures for various applications [48].

Our results confirm the conclusions of [16,17] that for the rectangular panels it is not rational to attach the square and round patches even at the central location of the defect. In the case under study, the patch of elliptical shape is the optimal one from the point of view of minimal loading of the repaired panel and patch. In practice, a serious limitation to the use of such patches, despite the minimal values of their loading factors, is the complexity of their manufacture [17]. As an alternative to the elliptical patch, an octagonal patch can be used provided that the shear stresses in the adhesive layer do not exceed the ultimate strength of the adhesive used. The insignificant difference (of about 3%) of the loading factors of the repaired panel and patch at the same area, and hence the weight, is compensated by the significant reduction of labour costs during its manufacture. 

The change in the patch dimensions and thickness has the opposite effect on the value of the loading factor of the adhesive interlayer between the panel and repair patch. The loading factors increase with the larger thickness of the patch and, conversely, decrease with the increase in its size. This confirms the conclusions of [46]. Therefore, in order to ensure the minimum mass of the repair patch, the choice of its thickness should be determined by the fulfilment of restrictions on the level of loading of the repaired panel and the repair patch, whereas the dimensions should meet the permissible stress level in the adhesive layer [17].

Increase in the number of steps of the repair lining leads to the more uniform distribution of the load between the elements of the repair joint. This increases the efficiency of use of the material. Therefore, the number of steps should be increased until an approximate equality of the values of loading factors of the repaired panel, patch and the adhesive interlayer between them is reached, or the possibilities for optimization of the shape of the repair patch cross-section are exhausted. It confirms the conclusions of [34,35]. At the same time, the larger amount of material necessary for the manufacture of the repair patch can be saved compared to that obtained in these papers. 

The proposed mathematical model is characterized by the certain area of applicability, where the accuracy of results obtained corresponds to the required one. Based on a comparison of the results with the data available in the literature [25,26], the error of the theoretical determination of deflection does not exceed 3%, and for stresses 5%. After the studies of stress–strain behaviour of the composite shell, minimum values of the degree of the polynomial describing the displacements *u_i_*, *v_i_* and *W*, were determined for different curvatures of the median surface. In accordance with our results, the values presented below were obtained at *N* = 12 for the case of asymmetric location of the notch and *N* = 6 for the symmetric problem. In the course of study of the flat plate, the convergence was satisfactory with *N* equal to 8 and 4, respectively, supporting the conclusions of [39]. To improve the accuracy of calculations, it is sufficient to increase the number of “mathematical” layers. However, this will lead to a significant increase in the number of variables and calculation time. 

The experimental method chosen for the study allowed, in contrast to the existing papers [7,43,44], us to obtain results in the form of the continuous displacement field. Therefore, we can gain a deeper understanding of the structure behaviour in the area of irregularity. Displacement fields available from experiments correspond to the predicted theoretical results. The resulting maximum error does not exceed 7% and demonstrates a good convergence of results. 

The choice of two fundamentally different types of finite elements for verification of one mathematical model is determined by its specific approach to the description of displacement and stress fields in the structure under study. On the one hand, in the analytical model a transition is made from the hypothesis of straight normals, underlying the calculations with the use of four-node multilayer four-angled shell elements, to the hypothesis of broken normals. It allows us to obtain more accurate results both for panels with individual layers of the low shear stiffness and for relatively uniform structures with a sharp variation in thickness [40,45]. On the other hand, displacements *U_i_* and *V_i_* (4–5) are, in fact, a linear interpolation of functions of two variables which determine the displacement fields in the interfaces between layers, instead of independent function of three variables typical for modelling with eight-node multilayer three-dimensional finite elements [41]. Therefore, comparison with the results obtained using these two types of finite elements allowed confirming the reliability of the model for determination of stress–strain behaviour of panels of step-variable thickness and assessing its rationality.

In general, theoretically obtained displacement and stress fields demonstrate good agreement in quality terms with the results of numerical modelling with different types of finite element. The difference in displacement values is 1.4% for deflection and 2.6% for in-plane displacements, reaching maximum values of 2.1% and 6.5% for the notched panel when modelling with multilayer 3D elements. For multilayer shell finite elements, these indices are 4.9 (9.9)% for deflection and 3.3 (11.6)% for in-plane displacements.

Summing up, we can draw a conclusion about the reliability of the mathematical model for calculating the stress–strain behaviour of the composite shell of step-variable thickness. Better convergence of theoretical results with the values obtained using multilayer three-dimensional finite elements confirm the need to use the refined theories in studies of structures of the considered type [28,29].

It should be noted that the mathematical model proposed in this paper to solve the problem allows taking into account the difference in the physical and mechanical characteristics of materials of the panel and repair patch, but does not allow simulating the rupture of the reinforcing fibres at the interface between the repaired panel and the patch. Therefore, assessment of the bearing capacity of the repaired structure should take into account the fact that the transfer of loads is carried out through the adhesive layer. According to a number of studies, additional safety factors are used to assess the bearing capacity of bonded structures [17,22].

## 7. Conclusions and Further Research

In accordance with the assigned task, a mathematical model to determine the stress–strain behaviour of an anisotropic shell of step-variable thickness, fixed in an arbitrary way, under the action of external loads and changes in the temperature field, was developed.

The results of experimental studies of the deformed state of a flat panel of step-variable thickness by the method of holographic interferometry demonstrate:

- correspondence of displacement fields obtained in practice to the predicted theoretical results;
- maximum deviation of the numerical values is less than 7%.

The number of full-scale tests insufficient to confirm the reliability of the developed model was supplemented by a comparison of analytical calculations with the results of modelling using the finite element method. Data obtained during modelling with two different types of finite element allowed us to draw a conclusion about the accuracy of theoretical calculations sufficient for engineering practice, and justify the need to use the refined theories to calculate the stress–strain behaviour of panels with sharp variation in thickness.

All this allows us to state that the proposed mathematical model for calculation of the stress–strain behaviour of anisotropic shells of step-variable thickness can be successfully used to solve practical problems such as determination of the stress–strain behaviour of a damaged structure or structure after repair, specification of the permissible delamination dimensions, and defining the parameters of the bonded repair process.

## Figures and Tables

**Table 1:** Results of calculation of the loading factors with the use of the repair patch of uniform thickness (Figure 10a).

$a^{\prime },\;\mathrm{mm}$	30	40	50	60	70	75	80
	$\delta^{\prime }$ = 0.2 mm						
$K_{0}$	1.56	1.56	1.55	1.50	1.43	1.42	1.41
$K_{p}$	1.28	1.42	1.49	1.41	1.30	1.29	1.28
$K_{ad}$	0.78	0.79	0.88	0.96	0.84	0.71	0.57
	$\delta^{\prime }$ = 0.4 mm						
$K_{0}$	1.13	1.13	1.12	1.06	0.98	0.97	0.96
$K_{p}$	1.17	0.79	0.84	0.76	0.66	0.65	0.64
$K_{ad}$	0.97	0.97	1.13	1.21	1.13	1.00	0.83
	$\delta^{\prime }$ = 0.6 mm						
$K_{0}$	0.86	0.87	0.86	0.79	0.72	0.70	0.70
$K_{p}$	0.75	0.68	0.57	0.48	0.40	0.39	0.38
$K_{ad}$	1.13	1.17	1.21	1.26	1.24	1.11	0.93

**Table 2:** Results of calculation of the loading factors with the use of the repair patch of the variable thickness with two steps (Figure 10b) at $\delta_{1}^{\prime }=\delta_{2}^{\prime }=0.1$ mm.

$a_{2}^{\prime },\;\mathrm{mm}$	55	60	65	70	75	80	85
$a_{1}^{\prime }$ = 85 mm							
$K_{0}$	1.02	1.01	0.99	0.97	0.96	0.96	0.95
$K_{p}$	0.73	0.71	0.69	0.68	0.68	0.67	0.67
$K_{ad}$	0.54	0.57	0.60	0.57	0.49	0.47	0.73
$a_{1}^{\prime }$ = 80 mm							
$K_{0}$	1.03	1.01	0.99	0.97	0.96	0.96	—
$K_{p}$	0.72	0.70	0.69	0.67	0.66	0.64	—
$K_{ad}$	0.66	0.69	0.68	0.58	0.53	0.83	—
$a_{1}^{\prime }$ = 75 mm							
$K_{0}$	1.03	1.01	0.99	0.98	0.97	—	—
$K_{p}$	0.71	0.70	0.68	0.66	0.65	—	—
$K_{ad}$	0.81	0.80	0.71	0.65	1.00	—	—
$a_{1}^{\prime }$ = 70 mm							
$K_{0}$	1.04	1.02	0.998	0.98	—	—	—
$K_{p}$	0.73	0.72	0.68	0.66	—	—	—
$K_{ad}$	0.90	0.81	0.74	1.14	—	—	—
$a_{1}^{\prime }$ = 65 mm							
$K_{0}$	1.06	1.03	1.02	—	—	—	—
$K_{p}$	0.77	0.73	0.70	—	—	—	—
$K_{ad}$	0.88	0.79	1.19	—	—	—	—
$a_{1}^{\prime }$ = 60 mm							
$K_{0}$	1.08	1.06	—	—	—	—	—
$K_{p}$	0.80	0.76	—	—	—	—	—
$K_{ad}$	0.83	1.18	—	—	—	—	—

**Table 3:** Results of calculation of the loading factors with the use of the repair patch of the variable thickness with three steps (Figure 10c) at $\delta_{1}^{\prime }=\delta_{2}^{\prime }=\delta_{3}^{\prime }=0.1$ mm.

$a_{3}^{\prime },\;\mathrm{mm}$	30	35	40	45	50
$a_{1}^{\prime }$ = 60 mm, $a_{2}^{\prime }$ = 50 mm					
$K_{0}$	0.87	0.86	0.86	0.85	0.84
$K_{p}$	0.62	0.57	0.54	0.55	0.54
$K_{ad}$	0.96	0.96	0.96	0.95	0.90
$a_{1}^{\prime }$ = 60 mm, $a_{2}^{\prime }$ = 40 mm					
$K_{0}$	0.88	0.87	0.86	—	—
$K_{p}$	0.62	0.61	0.60	—	—
$K_{ad}$	1.04	1.04	1.04	—	—
$a_{1}^{\prime }$ = 60 mm, $a_{2}^{\prime }$ = 30 mm					
$K_{0}$	0.87	—	—	—	—
$K_{p}$	0.79	—	—	—	—
$K_{ad}$	1.04	—	—	—	—
$a_{1}^{\prime }$ = 50 mm, $a_{2}^{\prime }$ = 45 mm					
$K_{0}$	0.91	0.90	0.89	0.88	—
$K_{p}$	0.68	0.64	0.61	0.62	—
$K_{ad}$	0.86	0.85	0.82	0.75	—
$a_{1}^{\prime }$ = 50 mm, $a_{2}^{\prime }$ = 40 mm					
$K_{0}$	0.91	0.90	0.89	—	—
$K_{p}$	0.68	0.67	0.67	—	—
$K_{ad}$	0.95	0.93	0.91	—	—
$a_{1}^{\prime }$ = 50 mm, $a_{2}^{\prime }$ = 35 mm					
$K_{0}$	0.91	0.89	—	—	—
$K_{p}$	0.70	0.70	—	—	—
$K_{ad}$	0.98	0.97	—	—	—
$a_{1}^{\prime }$= 50 mm. $a_{2}^{\prime }$= 30 mm					
$K_{0}$	0.90	—	—	—	—
$K_{p}$	0.76	—	—	—	—
$K_{ad}$	1.02	—	—	—	—
$a_{1}^{\prime }$= 40 mm, $a_{2}^{\prime }$= 35 mm					
$K_{0}$	0.92	0.90	—	—	—
$K_{p}$	0.75	0.81	—	—	—
$K_{ad}$	0.73	0.70	—	—	—
$a_{1}^{\prime }$= 40 mm, $a_{2}^{\prime }$= 30 mm					
$K_{0}$	0.90	—	—	—	—
$K_{p}$	0.85	—	—	—	—
$K_{ad}$	0.77	—	—	—	—
$a_{1}^{\prime }$= 30 mm, $a_{2}^{\prime }$= 30 mm					
$K_{0}$	0.86	—	—	—	—
$K_{p}$	1.06	—	—	—	—
$K_{ad}$	1.14	—	—	—	—

**Table 4:** Rational geometric parameters of the repair patches satisfying the criteria of operability of the repaired structure and the condition for the minimum mass.

Patch Parameters Characteristics of the Structure	Patch of the Uniform Thickness	Patch of the Variable Thickness	
		With Two Steps	With Three Steps
	$a^{\prime }=$ $\;75\;\mathrm{mm};\;\delta^{\prime }=0.4\;\mathrm{mm}$	$a_{1}^{\prime }$ $=70\;\mathrm{mm};\;a_{2}^{\prime }$ $=65\;\mathrm{mm};\;\delta_{1}^{\prime }=\delta_{2}^{\prime }=0.1\;\mathrm{mm};\;$	$a_{1}^{\prime }$ $=40\;\mathrm{mm};\;a_{2}^{\prime }$ $=a_{3}^{\prime }=$ $30\;\mathrm{mm};\;\delta_{1}^{\prime }=\delta_{2}^{\prime }=\delta_{3}^{\prime }=0.1\;\mathrm{mm}$
$\mathrm{Loading}\;\mathrm{coefficients}\;\mathrm{of}\;\mathrm{the}\;\mathrm{repaired}\;\mathrm{panel}\;K_{0}$	0.97	0.998	0.90
$\mathrm{Loading}\;\mathrm{coefficients}\;\mathrm{of}\;\mathrm{the}\;\mathrm{repair}\;\mathrm{patch}\;K_{p}$	0.65	0.68	0.85
$\mathrm{Loading}\;\mathrm{coefficients}\;\mathrm{of}\;\mathrm{the}\;\mathrm{adhesive}\;\mathrm{interlayer}\;K_{ad}$	1.00	0.74	0.78
Polymeric composite material amount for making a patch, dm^2^	22.5	18.25	6.8
Saving of polymeric composite material required for making a patch, %	—	18.9	69.76

**Table 5:** Comparative analysis of displacements.

Components of Displacement	Pressure *p* = 0.1MPa			Temperature ∆*T* = 100 °C		
	Theoretical Solution	Multilayer Shell Finite Element	Multilayer 3D Finite Element	Theoretical Solution	Multilayer Shell Finite Element	Multilayer 3D Finite Element
**Notched panel**						
*U*, mm	0.139	0.139	0.139	0.045	0.047	0.047
*V*, mm	0.025	0.023	0.027	0.0424	0.045	0.045
*W*, mm	−0.479	−0.529	−0.489	0.284	0.293	0.280
**Panel with a patch**						
*U*, mm	0.120	0.120	0.119	0.032	0.032	0.032
*V*, mm	0.005	0.006	0.005	0.027	0.028	0.027
*W*, mm	−0.438	−0.417	−0.443	0.213	0.206	0.210

**Table 6:** Comparative analysis of stresses.

Components of Stresses	Pressure *p* = 0.1MPa			Temperature ∆*T* = 100 °C		
	Theoretical Solution	Multilayer Shell Finite Element	Multilayer 3D Finite Element	Theoretical Solution	Multilayer Shell Finite Element	Multilayer 3D Finite Element
**Notched panel**						
${\left.\sigma_{\alpha }\right|}_{z=0},\;MPa$	47.32	54.69	52.22	42.10	48.25	46.87
${\left.\sigma_{\beta }\right|}_{z=0},\;MPa$	−15.26	−13.07	−13.89	−32.11	−31.93	−35.34
${\left.\sigma_{\alpha }\right|}_{z=1},\;MPa$	−15.91	−18.56	−17.05	−32.32	−31.78	−35.59
${\left.\sigma_{\beta }\right|}_{z=1},\;MPa$	−105.12	−91.90	−104.84	90.83	88.69	86.13
${\left.\tau_{\alpha \beta }\right|}_{z=0},\;MPa$	40.60	39.86	41.95	15.56	13.38	9.06
${\left.\tau_{\alpha \beta }\right|}_{z=1},\;MPa$	18.32	17.59	18.59	23.50	28.33	22.95
**Panel with a patch**						
${\left.\sigma_{\alpha }\right|}_{z=0},\;MPa$	64.43	54.20	64.36	38.84	32.47	40.09
${\left.\sigma_{\beta }\right|}_{z=0},\;MPa$	4.72	4.55	4.84	−10.81	−8.97	−11.61
${\left.\sigma_{\alpha }\right|}_{z=1.5},\;MPa$	29.06	24.65	31.23	−32.14	−31.92	−35.37
${\left.\sigma_{\beta }\right|}_{z=1.5},\;MPa$	−4.24	−3.79	−4.15	−24.65	−28.59	−24.19
${\left.\tau_{\alpha \beta }\right|}_{z=0},\;MPa$	38.02	37.51	39.09	17.49	15.11	10.27
${\left.\tau_{\alpha \beta }\right|}_{z=1},\;MPa$	17.99	17.61	17.95	24.18	29.66	24.03

## Data Availability

Not applicable.
